# The COVID‐19 Pandemic—Can open access modeling give us better answers more quickly?

**DOI:** 10.1002/acm2.12941

**Published:** 2020-06-01

**Authors:** Mary Beth Allen, Michael Mills, Mehdi Mirsaeidi

**Affiliations:** ^1^ School of Public Health University of Louisville KY USA; ^2^ Division of Pulmonary and Critical Care University of Miami Miami FL USA

The coauthors, Mary Beth Allen, MBA, PhD and Mehdi Mirsaeidi, MD, MPH, are my former colleagues from the University of Louisville and both have significant experience and expertise regarding infectious disease. Dr. Allen is a healthcare research consultant and former senior program manager for infectious disease at the University of Louisville. Dr. Mirsaeidi is currently an Associate Professor at the University of Miami Miller School of Medicine—Michael Mills.

There are many dimensions to an emergent pandemic such as COVID‐19. Leaders all over the world are grappling with the complexity of this disease as they consider the multifaceted burden it places on communities, healthcare delivery systems, financial security, and the role of government. Effective policy must address each and every dimension of this pandemic and do so early and decisively while also earning public confidence in order to ensure cooperation and compliance. While some policy makers are adept at the formation, implementation, and assessment of public policy, many rely on a myriad of resources for support through complex problems. Among these resources are the support of experts representing various dimensions of the problem situation, historical data from previous pandemics, observational data from other impacted regions, and the use of systems dynamics tools that predict the spread and impact of disease over time. System dynamics describes the process of representing a complex system with interrelated parts that interact in a nonlinear and unpredictable method within a system and predicting those interactions and outcomes. The process of mapping a system is described as system thinking, and this step alone can force its users to easily understand and predict outcomes in a complex system using mental modeling. The next step applies the map into a mathematical model through a system of algorithms to calculate the interactions and make predictions regarding future interactions and outcomes over time. System dynamics has many applications for both routine and unexpected problem situations and represents an important decision support tool that helps make determinations about the effectiveness of potential policy interventions prior to implementation. Modeling as a resource not only has many implications for the current pandemic, but also may play an important role in the dynamic field of medical physics.

Modeling has played a key role in the management of the COVID‐19 pandemic in many settings. By predicting the epidemiology of this disease, modeling has allowed policy makers, healthcare delivery systems, and other stakeholders to make short‐ and long‐term policy that addresses the emerging needs of systems on the local, state level, and national level. However, effective modeling is dependent upon accurate data along with the integration of valid assumptions. For this situation specifically, the rapid accumulation of data being generated worldwide has the potential to support better modeling, and making this timely data available for immediate use through open source publishing could be a huge benefit. With so many unknowns still associated with this particular coronavirus being a novel virus, any accumulation of data is key as so many questions remain regarding diagnostics, risk factors, disease presentation, transmission patterns, and treatment.

Access to timely data is also key as enacting policy early in a pandemic is an important component of its impact and outcomes. When it was clear that the virus had arrived to the US, it was incumbent on national and state leaders to act swiftly and decisively. Because limitations in the ability to administer adequate testing and isolate early cases left policymakers with few options, widespread social distancing became state and national policy that led to forced closures of major sectors of the economy. These policies had an immeasurable impact on the economy forcing immediate and record job loss, market volatility, and uncertainty regarding the future of many industries. While concerns for the economic system that provides financial security for so many remains an important consideration, American economists with few outliers have supported lockdowns as a means mitigating the loss of life and other burdens of disease that can have a lasting impact on the economy. Modeling using accurate data can not only have an impact on predicting the epidemiology of disease, but it can also be used to address other dimensions of this pandemic that may be just as critical to the health and safety of Americans.

Still, the vast majority of models have focused specifically on the epidemiology of disease. Several models predicting the epidemiology in the US have been released during different time periods in this pandemic that have driven national policy. The increased availability of data, changes in state policy and local responses, and access to new technology will continue to impact the outcomes of these models, which depend on timely information from worldwide sources. Among the earliest models released, a March 17 report from epidemiologist Dr. Neil Ferguson published by the Imperial College of London under the umbrella of the World Health Organization predicted that mortality for COVID‐19 at 2.2 million people in the US. This model relies heavily on data from the 1918 H1N1 pandemic, assuming a R_0_ of 2.4 with symptomatic individuals 50% more contagious than asymptomatic individuals, an incubation period of 5.1 days, immunity following infection, and no widespread and enforced social distancing policy enacted (https://www.imperial.ac.uk/media/imperial‐college/medicine/sph/ide/gida‐fellowships/Imperial‐College‐COVID19‐NPI‐modelling‐16‐03‐2020.pdf). In contrast, models released by the US Coronavirus Task Force have provided much lower numbers, but each of these models has only calculated mortality as an outcome. The first model was released on March 30 and anticipated 200 000 deaths in the US. The only assumption that was shared as a basis for these predictions was strong and effective policy interventions nearing perfection in terms of adherence and reaching goals (https://www.washingtonpost.com/national/coronavirus‐deaths‐warning‐america/2020/03/30/522221ce‐72a6‐11ea‐87da‐77a8136c1a6d_story.html). Several updates to this initial model have been reported, the first of which was released on April 1 noting a much lower projected mortality of 90 000 (https://www.latimes.com/politics/story/2020‐04‐08/new‐data‐suggests‐u‐s‐deaths‐may‐be‐lower‐than‐feared), mortality at 60 415 through August 4, 2020 reported on April 8 (https://covid19.healthdata.org/united‐states‐of‐america), and rising to a range between 80 000 and 90 000 deaths with daily mortality of 3000 through June 1, (https://www.nytimes.com/2020/05/04/us/coronavirus‐updates.html). A May 4, 2020 updated model increased anticipated deaths to 134 000 in the US as a result of the relaxation of state‐level social distancing policies (https://www.cnn.com/2020/05/04/health/us‐coronavirus‐monday/index.html). This number was most recently increased to 147 000 by early August on May 12, 2020 (https://www.forbes.com/sites/mattperez/2020/05/12/coronavirus‐model‐used‐by‐white‐house‐now‐projecting‐147000‐us‐deaths‐by‐early‐august/#6c259abcb0f1). Among the major assumptions in these latest models is the relaxation of social distancing and the reopening of major segment of the economy as is occurring in most every state.

The utility of models extends well beyond generating an accurate prediction, as it is well understood that all models have their limitations. Models test the effectiveness of policy or calculate the rate of change in a variable, and they do so to provide support for policy. Among the limitations of these models used on the federal level is that the myriad of embedded assumptions, variables, and mathematical process used to build the model have not been made available. This has huge implications for the validity of the model, while also acting to exclude collaboration from experts within the academic and scientific community. The sharing of resources and information promotes the creation of better research, processes, policy, and outcomes. Also, the majority of these models only measure the outcome of mortality, as other key outcomes such geographical spread, number of cases, and hospitalizations have important implications for state and community level policy and resource planning. Furthermore, the timeliness of the data used as inputs for these models are unknown, but there is no question that immediately available open source could be an asset for this rapidly changing problem situation.

Using a systems dynamics approach to modeling and understanding the rapid spread of an infectious disease, such as COVID‐19, researchers from the University of Alicante, Spain created an open access model that is sufficiently complex, adaptable, and potentially valuable to policymakers all over the world. The publication provides an open access model published on the STELLA® platform (iseesystems), a widely adopted platform that allows users to customize the model to any area, and evaluate the behavior of the disease using a wide variety of projections, measured data, and scenarios. Although this publication is not currently peer reviewed, users of this technology will immediately appreciate the complexity and sophistication of the model and its potential to yield results that are meaningful and a source of significant utility. Additionally, since all components of the model may be viewed and are publicly available, the time to develop consensus and wide political support could be accelerated. As many regions and communities adopt the model, the data has the potential to be aggregated and applied as important policy support for major transitions in public policy (https://www.medrxiv.org/content/10.1101/2020.03.30.20047043v1).

Among the variables of the model, key inputs include the total population, the number infected, the number of contacts per day (R_0_), the rate to symptomatic presentation, the asymptomatic rate, the rate of patients hospitalized, and the rate of ICU patients hospitalized. The R_0_ is not a single number, but a graphical function over time, which is likely much more realistic and allows for more accurate predictions that calculate the impact of social distancing policy over time, as well as the impact of relaxing those measures. With accurate information as inputs, this model is capable of predicting not only mortality, but also other key outputs such as peak curves, case count, and hospitalization. The article uses official data from the Spanish Ministry of Health as input for calibration (https://www.mscbs.gob.es/en/profesionales/saludPublica/ccayes/alertasActual/nCov‐China/home.htm).

Although this model has important utility it is likely less complex than models used by major governments, international organizations, and prestigious academic institutions. For this reason, it is well suited for regional and community level adoption, which in the US remains the governmental institution involved in creating and enforcing pandemic response policy. Like all models, the utility will improve as the variables used as inputs become more accurately measured or defined. In the US, this will depend on the continued availability of diagnostic testing in both the outpatient and hospital settings along with accurate antibody testing. Also, public health functions and continued surveillance such as contact tracing might result in much more accurate numbers, including better estimates of R_0_ over time. The authors are careful to remind readers that models are inherently wrong and exist less as a means of predicting outcomes, but more as a utility for policy makers. The authors also emphasize their open access format as an important strength, which makes this resource available to potentially anyone as an adaptable resource that can be applied to a variety of settings. Although some familiarity with STELLA software is required to adapt the model, this should not be a major challenge for those comfortable with other modeling programs. The authors are currently developing a video tutorial as a resource to support the adaption of this model in order to address any specific barriers to this software. Also, the authors of this paper feel strongly about the importance of social distancing policy relative to the COVID‐19 pandemic, encouraging that this policy be maintained over time. This recommendation stands despite skeptics who erroneously attribute this policy to undue economic hardship. Sweden became an interesting test case for an alternative to forced closure of the private business, and the outcomes were a case mortality that was remarkably higher than similar populations (https://www.spiked‐online.com/2020/04/22/there‐is‐no‐empirical‐evidence‐for‐these‐lockdowns/). But, the evidence that social distancing policy had reduced transmission in many settings continues to accumulate. A study conducted at the University of Kentucky found that the aggressive Healthy At Home initiative, which has resulted in Kentucky ranking among the highest in the US in terms of Coronavirus response, is believed to have saved as many as 2000 lives in the commonwealth so far, with less than 200 deaths having been reported at that time (https://www.kentucky.com/opinion/linda‐blackford/article242367161.html).

The need for modeling will continue to be an important resource as new data continues to emerge and social distancing policies relax across the world. Also, the experiences from different settings will become data points in an algorithm. Communities in Italy and Spain were among the earliest impacted regions in Europe, but the late to arrive the UK has currently surpassed these early hot spots in terms of case count and mortality despite having more data. Meanwhile, Germany over prepared with early lock downs, initiated widespread testing, and an excess of hospital capacity shared with its neighbor by taking Italian patients. In the US, New York was the epicenter for the American burden with communities in Florida and Illinois on the rise. Also, state leadership of lockdown policy has resulted in a patchwork of lockdowns targeting different industries with different timelines for reopening, which will provide rich data for analysis. Creating a mechanism to share this data in real time through open source publishing could make this analysis available sooner to support the evaluation of current policy rather than historic analysis.

Medical science depends on the proliferation, dissemination, and collaboration of knowledge. Increasing the availability of timely data though open source publishing has the potential to escalate this process, which has the potential to benefit everyone. This process begins with individual sectors of the medical community making available to one another and related sectors valuable and emerging research that is relevant to the practice and the evolution of a discipline. Also, despite the specialization and fragmentation of medicine, major developments from one discipline can have a resounding impact on many other fields of practice. Furthermore, resources developed in related fields and from industry have found adoption in the field of medical physics. System dynamics modeling is no exception as it has been adopted specifically in the economics of medical physics, and has been noted to predict the supply and demand of radiation oncology physicists with accuracy (https://aapm.onlinelibrary.wiley.com/doi/10.1120/jacmp.v11i2.3005). In regards to the COVID‐19 pandemic, it is safe to state that no sector of the economy, much less no sector of the medical economy, will remain insulated from the impact likely for years to come. As modeling may predict the distribution and outcomes of disease in care settings where we practice, we will be able to adapt these models in a variety of ways as our roles have evolved substantially within hospitals, universities, and our communities. The unique disease curve for each of us will not be congruent, and we too can use modeling to make calculations regarding the needs for policy interventions relative to our unique care delivery systems. This might be especially critical for our medically fragile patients who will depend on us to provide for their safety. For this reason alone, we have a responsibility to anticipate challenges to the healthcare system and communicate recommended interventions to our elected leaders, hospital administrators, and other important community level leaders. By acting early, we can be not only supporting our patients, but also acting in support of our leaders and our communities. We are also obligated to protect ourselves as essential healthcare workers and our employees who act in support of essential care delivery. Identifying our own risks and exposures, creating policy to undermine that risk, and calculating the optimal timing of changes in policy are all important interventions we can make on the community level that can be supported by modeling. Furthermore, as the current crisis concludes, perhaps the adoption of modeling skills can serve our field in the future. Systems dynamics can be used to model daily practice and its many disruptions, which could include minor changes in reimbursement or payer mix or the impact of new technology as is common in our profession. If we gain the ability to adapt systems dynamics, there is no end to the applications of this technology as well as the daily application of systems thinking, which describes a mental model that emulates the technology by analyzing and predicting the interaction of interrelated parts of a system over time. Healthcare delivery is dynamic, but the technology rich and constantly evolving field of medical physics is arguably among the most dynamic in all of healthcare, which implies that systems dynamics may have an important role in the field of medical physics. As we remain collaborative with other fields, most notably though the proliferation of open access formats becoming more common in other fields of medicine, basic science, social science, and industry, it is likely that systems dynamics may represent one of several opportunities for medical physics to advance.

